# Pure ultrasonography-guided radiation-free percutaneous nephrolithotomy: report of 357 cases

**DOI:** 10.1186/s40064-015-1078-4

**Published:** 2015-07-03

**Authors:** Mohammad Mehdi Hosseini, Alireza Yousefi, Mohsen Rastegari

**Affiliations:** Shiraz Nephrology-Urology Research Center, Shiraz University of Medical Sciences, Shiraz, Iran; Urology Department, Jahrom University of Medical Sciences, Jahrom, Islamic Republic of Iran; Minimal Invasive Unit, Urology Department, Shiraz University of Medical Sciences, Shiraz, Iran

**Keywords:** Renal calculi, Percutaneous nephrolithotomy, Nephrolithiasis, Ultrasound

## Abstract

**Purpose:**

To assess the safety and effectiveness of pure ultrasound-guided percutaneous nephrolithotomy.

**Patients and methods:**

Three hundred fifty-seven patients were treated; 139 women and 218 men, with a mean age of 33.7 years (range 21–69 years) and a mean stone size of 33.5 mm in maximum diameter (range 20–52 mm). Stone locations were renal pelvis (174), lower calyx (68) or both (115) with mild to moderate hydronephrosis seen on excretory urography. A ureteral stent was inserted by cystoscope, and saline was injected for better localization of the pelvicaliceal system (PCS), if needed. Puncture of the PCS was done by an 18-gauge nephrostomy needle through the lower pole calyx, and all the steps, including dilatation, were done under the guidance of ultrasonography.

**Results:**

The day after the operation, 318 (89.07%) patients were stone-free in the kidneys, ureters, and bladder x-rays. Nineteen patients (5.3%) had multiple fragments that measured equal or less than 5 mm and passed them spontaneously in 2–4 weeks (total stone-free rate 94.4%). Access failure occurred in ten obese patients (2.8%) and fluoroscopy was required. Residual fragments with sizes of 10–12 mm were seen in seven patients, all of who underwent shock wave lithotripsy. In one patient, a fragment measuring 7–8 mm migrated into the distal part of the ureter. It was fragmented with ureteroscopy and pneumatic lithoclast 2 days after the operation. In two patients who had large (>15 mm) residual stone redo percutaneous nephrolithotomy was performed 48 h after the first procedure.

**Conclusion:**

Percutaneous nephrolithotomy guided by ultrasonography seems to be as effective as fluoroscopy in selected cases and poses no risk of surgeon and patient exposure to radiation; however, more experience is required.

## Background

Various methods have been used to manage renal stones. Of them, percutaneous nephrolithotomy (PCNL) is specifically very effective to remove large renal stones (Tiselius et al. 2001). It is a minimally invasive procedure through which the access to the pelvicalyceal system (PCS) is achieved by the puncture and dilatation of the tract under fluoroscopic guidance. This step is an important one that can significantly affect the outcome of the procedure (Osman et al. 2005; Michel et al. 2007). However one of its major disadvantages is the risk of exposing the operators and patients to radiation. To avoid this disadvantage ultrasonography (US) has been used for PCNL in some studies, which showed some advantages (Hacker et al. 2007; Mozer et al. 2007; Marino et al. 2002; Hosseini et al. 2009). It has no risk of radiation hazard while can provide visual information about other body organs such as liver and spleen. In this study we aimed to report our experience of managing large renal stones in our center using US-guided PCNL.

## Patients and methods

From August 2003 to April 2014, all patients with US-guided PCNL were registered into the study (Table 1). Patients with staghorn stone or calyceal diverticular stones as well as those with non-dilated PCS or renal anomaly were excluded. Renal pelvis and lower pole seems more easy for successful access and stone free state by ultrasound guidance. Patients were evaluated preoperatively by imaging studies, including plain abdominal radiography, ultrasonography, and excretory urography (EXU). The advantages and disadvantages were discussed, and the possibility of premature termination or multisession PCNL was explained to each patient before surgery. Preoperative care included light bowel preparation and a single intravenous dose of ceftriaxone.

**Table 1:** Demographics of patients who underwent USG-PCNL (357)

	Number	Percent (%)
Male/female	218/139	61/39
Right/left	184/173	51.5/48.5
Mean age (year)	33.7 ± 11	
Mean stone size (mm)	33.5	
Stone location		
Renal pelvis	174	48.7
Lower pole	68	19
Pelvis and lower pole	115	32.2
Co-morbidities		
Diabetes	17	4.8
Hypertension	27	7.6
Heart diseases	21	5.9
Obesity	15	4.2
Renal insufficiency	7	2

### Surgical technique

The US-guided PCNL technique has been explained elsewhere (Hosseini et al. 2009). Briefly, the patient is placed in the lithotomy position and, under general or epidural anesthesia, a 6 Fr ureteral catheter is passed into the ureter and kidney by cystoscope. After that the patient is repositioned in the prone position, and percutaneous access to the lower pole of the kidney is obtained under US guidance. It is safe and simple path for access to the lower pole and renal pelvis stone, however, for staghorn stones upper pole, and for pelvis stone, mid-pole calyx were recommended for access to all PCS, ureteropelvic junction and proximal ureter. Retrograde instillation of saline into the PCS was used for enhancing the degree of hydronephrosis and better visualization of the PCS. We used colour-Doppler US system with a 3.5-MHz probe (BK Medical). At first, US was performed for detection of possible organs located between the puncture site on the skin to the kidney to prevent injury. Under guidance of the US, the lower calyx was punctured with an 18-gauge nephrostomy needle. Then its stylet was removed and after draining urine, J-tip wire was sent into the PCS. An incision about 10–12 mm was made alongside the sheath of needle and got out it. We measured the length of the needle from the skin to PCS for estimation of dilators length. The antenna of the metallic telescopic dilator of the Wolf system was sent into the PCS on the wires. The tract was dilated up to 28 Fr in one step with cone-tip plastic or metallic dilator, and the Amplatz sheath was placed in the PCS. Nephroscopy was performed with a 24 French Wolf nephroscope and the stone was fragmented by pneumatic lithotripter (Lithoclast, EMS). The stone fragments were removed by forceps. In 11 cases who had solitary kidney, antegrade JJ stent was inserted and ureteral catheter was removed. Finally, the presence of any residual stones was checked with US. Floroscopy-guided PCNL was performed in cases in whom USG was failed.

### Study outcomes

All patients’ demographic characteristics, including age, sex, and stone size were recorded. Intraoperative data such as operative time, intraoperative difficulties, and complications were also noted. To assess blood loss and changes in renal function during surgery, preoperative and 6-h postoperative hemoglobin (Hb) and creatinine concentrations were recorded and compared. Likewise, major postoperative complications and any need for ancillary procedures were recorded. The presence and size of any residual stones were recorded by KUB radiography and ultrasound at the first day, 1 month after the operation, and then at regular 3-month clinical follow-up visits.

## Results

Of 357 cases, stones were located in renal pelvis in 174 (48.7%), lower pole calyx in 68 (19%), and both pelvis and lower pole in 115 patients (32.2%).Three hundred eighteen (89.07%) patients were stone-free in the postoperative KUB/US the day after the procedure. Nineteen patients (5.3%) had multiple fragments that measured about 5 mm or less and passed them spontaneously in 2–4 weeks (final stone-free rate: 94.4%). Access failure occurred in ten obese patients (2.8%), and fluoroscopy was required. Seven patients (1.96%) had residual fragments that measured l0–12 mm and underwent shock wave lithotripsy. In one patient, a fragment that measured 7–8 mm migrated into the ureter. It was fragmented and removed with ureteroscopy 2 days after the operation. In two patients who had intraoperative hemorrhage, the procedure terminated and nephrostomy tube was inserted. They underwent second look operation 48 h after the first procedure.

Access-related complications, such as bending of the guide-wire, visceral organ injury, and perinephric collection, were not reported in any patients. Fourteen patients (3.9%) required blood transfusions, and 26 (7.3%) experienced fever that was resolved with conservative therapy.

Headache developed 4–5 days after the operation in 17 patients (4.8%) who had received epidural anesthesia for their surgery. All were managed conservatively with analgesics. Severe post-operative renal colic was observed in 8 (2.3%) patients which was managed conservatively in five patients and with ureteral stenting in three patients. There was no major intra-or postoperative complications (Table 2).

**Table 2:** Results and complications of USG-PCNL (357)

	Number	Percent (%)
Stone-free rate	337	94.4
Mean operation time (min)	65 (40–85)	
Mean access time (min)	6.4 (4–11)	
Access failure	10	2.8
Residual stone	9	2.5
Ancillary procedure		
ESWL	7	2
URS	1	0.3
Redo PCNL	2	0.6
Transfusion	14	4
Fever	26	7.3
Headache	17	4.8
Renal colic	8	2.3
PCS perforation	15	4.2

*ESWL* extracorporeal shockwave lithotripsy, *URS* ureteroscopy, *PCS* pelvicalyceal system.

## Discussion

Currently the most common method to treat large or complicated renal stones that cannot be managed by shock wave lithotripsy (SWL), is PCNL (Karami et al. 2010). To perform the PCNL, proper PCS puncture is a crucial step. Using fluoroscopy during this method is accompanied by exposing the operators including the surgeons as well as the patients to radiation. Such radiations may lead to hazardous effects on various organs including the gonads, bone marrow, eyes, and thyroid (Gamal et al. 2011). Of the organs, gonads are more sensitive as spermatogonia in the male testis are highly affected by the radiation. It has been reported that even a small dose of 0.15 Gy can lead to low sperm count. And if the radiation increases to more than 0.5 Gy, transient azoospermia may occur. Sterility has also been reported by exposure to high doses of radiation (5–6 Gy) (Hall and Giaccia 2006).

Access to the PCS can be done by using fluoroscopy, US, or a combination of both techniques. The best tract to be approached is the direct path, which is from the skin through the papilla of the desired calyx and renal pelvis (Agarwal et al. 2011). This approach can be performed by using either the fluoroscope or US. However of the advantages of using US is guiding the puncture needle to the posterior calyx without any considerable complications (Gupta et al. 1998). US is also the preferred option for those patients for whom cystoscopy and/or ureteric catheterization are impossible. It is also a good option for pregnant women who are scheduled for PCNL (Agarwal et al. 2011). Osman and colleagues reported 315 PCNLs guided by US that they performed during 1987–2002. They punctured the PCS under the US guidance, but the dilation of tract was guided by fluoroscopy, while the patients were in a prone position. They reported 50.8% complication rate, although most of them were not clinically significant. They achieved 96.5% stone-free rate using the US-guided renal access (Tiselius et al. 2001). Desai and co-workers reported the advantages of using US to puncture PCS. They also believed that this techniques not only can help surgeon to avoid radiation, but also visceral injury, and intra-renal vascular damages can be prevented too. They concluded that straight access toward the PCS, which can be achieved using US may lead to lower morbidity (Desai 2009).

Etemadian and colleagues assessed the safety and efficacy of transcutaneous nephrolithotomy guided by US in 12 patients during December 1999–December 2000. Of the patients, 11 patients were stone-free after 3 months. They recommended using US in patients whose PCS were dilated and had large stones in renal pelvis or calyx. (Etemadian et al. 2004)

We have also documented the feasibility, reliability, safety, and effectiveness of US-guided PCNL over fluoroscopy in a study done from August 2003 to December 2007 (Hosseini et al. 2009). Basiri and colleagues reported their experience of performing PCNL guided by US in 30 patients who were in flank position. They reported 88.9% stone-free rate in patients who had a single calculus and 75% in patients who had staghorn or multiple calculi. The researchers concluded that US-guided PCNL could have acceptable outcomes without considerable complications in comparison with the standard technique (Basiri et al. 2008a).

In another study, Basiri and colleagues reported the results of their trial on 100 patients without upper urinary tract abnormalities. They concluded lower exposure to radiation and access duration in patient who underwent PCNL guided by US compared with those received fluoroscopy-guided access (Basiri et al. 2008b). In another randomized trail on 40 patients, Karami and co-workers showed successful US-guided PCNL in all their patients who were positioned in lateral decubitus flank. They reported safety and efficacy of US-guided PCNL as their complete stone-free rate was 85%. They suggested using this technique in the lateral decubitus flank position to prevent hazardous effects of radiation to patients, surgeons, and surgical team (Karami et al. 2009). Agarwal and others also reported shorter mean time for successful puncture and radiation exposure in their patients who underwent puncture guided by US. Their technique yielded complete stone clearance with no considerable morbidity (Agarwal et al. 2011). In another study 100% successful access to PCS was reported by Gamal and colleagues. Their approach to the PCS was through the most convex point to prevent vascular damage; hence no intraoperative bleeding was reported. Only two cases of PCS perforation (5.9%) during dilatation by metal dilators was reported, which could be resulted from the difficulty in simultaneous monitoring of the metal dilator and the tip of the central metallic bar (Gamal et al. 2011). In a study on 92 patients, Zhou and colleagues reported the need for a second tract in 22.8% of their patients. They mentioned that the reason for their finding was that about two-thirds of their patients had multiple and staghorn stones (Zhou et al. 2008).

Song Yan and co-workers reported 705 cases of PCNL by pure sonography. They showed 92.6% stone-free rate in single stone and 82.9% in staghorn or multiple cases (Yan et al. 2013).

In our study, the total stone-free rate was 94.4%, with no significant morbidity (Table 2). Access failure occurred in ten morbidly obese patients for whom we had to use fluoroscopy. In 15 patients the PCS was perforated (4.2%) during dilatation, which is in accordance with the report by Gamal and colleagues who reported perforation in two (5.9%) patients.

Although US-guided PCNL has many advantages, such as the high success rate for PCS puncture, intra-operative detection and monitoring of both radiolucent and radiopaque stones, lower risk of radiation exposure, and the ability to monitor all organs in the path of the puncture; it should be considered that it is an operator-dependent procedure, and to perform PCNL guided by US, sufficient experience must be acquired before routine performance. We suggest the use of fluoroscopy in difficult cases. When US-guided PCNL is applied for patients with opaque stones, a single visualization with fluoroscopy at the end of the procedure can be beneficial for determining the final stone-free rate.

## Conclusion

US-guided PCNL has satisfactory outcomes without any major complications compared with the standard fluoroscopy-guided technique. It has some advantages, such as prevention of radiation exposure and adjacent organ damage. It is also an efficient method for localizing both radio-opaque and radiolucent renal stones for patients with failed retrograde ureteral stenting as well as pregnant women. It should be considered that fluoroscopy guided PCNL is the standard method especially in training center. In high volume centers and with sufficient experience US-guided PCNL may be a good alternative for reducing radiation exposure of the patient and surgical team.
